# *Lactococcus petauri* sp. nov., isolated from an abscess of a sugar glider

**DOI:** 10.1099/ijsem.0.002303

**Published:** 2017-09-25

**Authors:** Laura B. Goodman, Marie R. Lawton, Rebecca J. Franklin-Guild, Renee R. Anderson, Lynn Schaan, Anil J. Thachil, Martin Wiedmann, Claire B. Miller, Samuel D. Alcaine, Jasna Kovac

**Affiliations:** ^1^​ Department of Population Medicine and Diagnostic Sciences, Cornell University, Ithaca, NY 14853, USA; ^2^​ Department of Food Science, Cornell University, Ithaca, NY 14853, USA; ^3^​ North Dakota State University Veterinary Diagnostic Laboratory, ND, USA; ^†^​ Present address: Washington Animal Disease Diagnostic Laboratory, Pullman, WA 99164, USA.; ^‡^​ Present address: Department of Food Science, Pennsylvania State University, State College, PA 16802, USA.

**Keywords:** *Streptococcaceae*, *Lactococcus*, Marsupialia, genome, aquaculture, farms

## Abstract

A strain of lactic acid bacteria, designated 159469^T^, isolated from a facial abscess in a sugar glider, was characterized genetically and phenotypically. Cells of the strain were Gram-stain-positive, coccoid and catalase-negative. Morphological, physiological and phylogenetic data indicated that the isolate belongs to the genus *Lactococcus*. Strain 159469^T^ was closely related to *Lactococcus garvieae* ATCC 43921^T^, showing 95.86 and 98.08 % sequence similarity in 16S rRNA gene and *rpoB* gene sequences, respectively. Furthermore, a pairwise average nucleotide identity blast (ANIb) value of 93.54 % and *in silico* DNA–DNA hybridization value of 50.7  % were determined for the genome of strain 159469^T^, when compared with the genome of the type strain of *Lactococcus garvieae*. Based on the data presented here, the isolate represents a novel species of the genus *Lactococcus*, for which the name *Lactococcus petauri* sp. nov. is proposed. The type strain is 159469^T^ (=LMG 30040^T^=DSM 104842^T^).

Sugar gliders (*Petaurus breviceps*) are small marsupials native to Australia and New Guinea and commonly kept as pets. Due to a specialized dental structure that is designed for peeling bark and not for eating soft diets, they are prone to oral cavity disease as companion animals; abscesses due to facial trauma are also common [1, 2]. Sugar gliders are susceptible to a number of bacterial and parasitic infections including *Pasteurella multocida* and *Toxoplasma gondii* [3], but are typically not associated with infections caused by members of the genus *Lactococcus*.

The genus *Lactococcus* is a member of the family *Streptococcaceae* composed of lactic acid fermenters. Members of this genus are commonly used in food production, especially in the dairy industry. *Lactococcus garvieae*, originally isolated from a mastitic cow udder [4], is a common pathogen of fish that is often isolated from environmental sources such as farm animal bedding. Lactococcosis is a serious concern for the global aquaculture industry. Although it rarely causes gastrointestinal disorders and infective endocarditis in humans [5, 6], *L. garvieae* has been described as an emerging zoonotic pathogen [7, 8].

Strain 159469^T^ was the predominant bacterial strain isolated from a facial abscess swab on a sugar glider submitted for routine clinical culture. The colonies had a distinctive bright orange pigment (Fig. S1, available in the online Supplementary Material). The strain was initially typed as a representative of *L. garvieae* based on biochemical profiles obtained from commercial diagnostic platforms and 16S rRNA gene Sanger sequencing. The pigmentation of the colonies, however, was inconsistent with this identification, and further characterization by whole-genome sequencing was performed. Phylogenetic and phenotypic analyses subsequently indicated that this strain represents a distinct species of the genus *Lactococcus*, sharing most of its sequence in common with *L. garvieae* and the pigmented phenotype of *Lactococcuslactis*. We propose to name 159469^T^ as the type strain of *Lactococcus petauri* sp. nov.

A 1537 bp 16S rRNA gene sequence was extracted from an assembled draft genome of isolate 159469^T^ using RNAmmer 1.2 [9] and checked for the presence of chimera using decipher [10]. NCBI blast identified the 16S rRNA gene sequence from *L. garvieae* strain M14 as the closest match to the 16S rRNA gene sequence of isolate 159469^T^. Comparative phylogenetic analysis was carried out with 16S rRNA gene sequences of isolate 159469^T^ and type strains of 16 species and subspecies of the genus *Lactococcus* with validly publsihed names. Strain 159469^T^ clustered close to *L. garvieae* JCM 10343^T^ (=ATCC 43921^T^) in a maximum-likelihood tree reconstructed on the basis of 16S rRNA gene sequences in RAxML v. 8 using the general time-reversible (GTRGAMMAI) substitution model and 1000 bootstrap repetitions (Fig. 1; [11]). *L. garvieae* JCM 10343^T^ was confirmed as the closest relative of strain 159469^T^ based on the 95.86 % sequence similarity of aligned 16S rRNA gene sequences (muscle; [12]). The 16S rRNA gene sequence identity of <97 % [13] suggested that isolate 159469^T^ represents a novel species. The same alignment was used to reconstruct the neighbour-joining (Fig. S2) and maximum-parsimony trees in mega (Fig. S3) [12]. All three tree reconstruction methods produced congruent clustering of *L. petauri* sp. nov. 159469^T^ with *L. garvieae* JCM 10343^T^, *L. garvieae* subsp. *bovis* BSN307^T^ and *Lactococcusformosensis* NBRC 109475^T^.

**Fig. 1.**
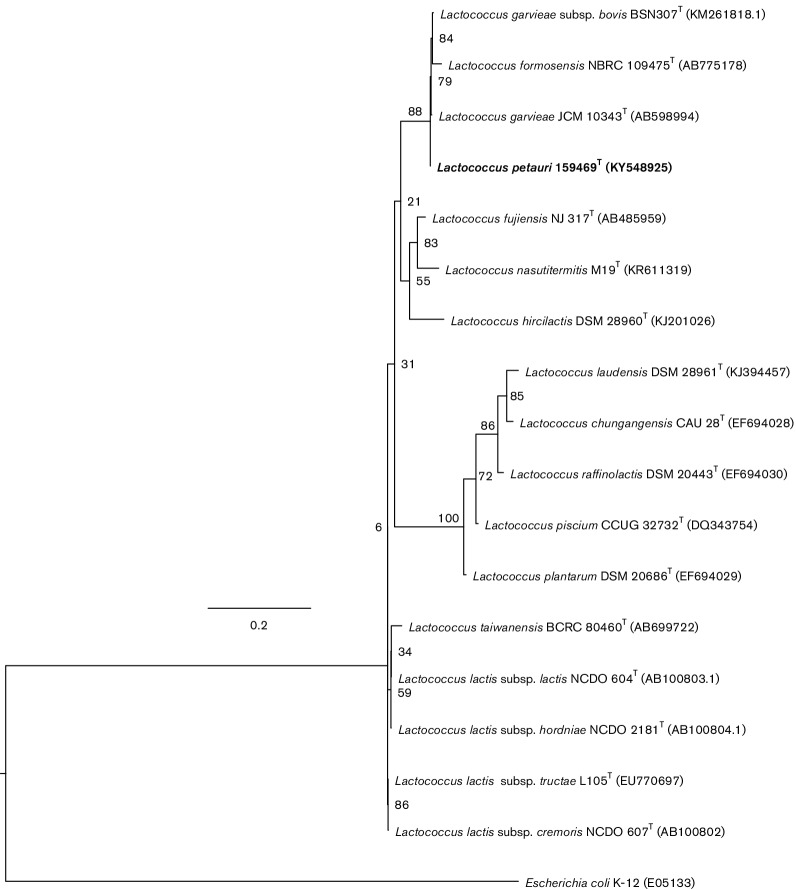
Rooted 16S rRNA gene maximum-likelihood tree of *Lactococcus petauri* sp. nov. 159469^T^, 16 type strains of species and subspecies of the genus *Lactococcus*, and *Escherichia coli* K-12 as an outgroup. The tree was reconstructed in RAxML v. 8, using the GTRGAMMAI substitution model and 1000 bootstrap repetitions. Numbers at nodes indicate percentage bootstrap support. Bar, 0.2 substitutions per site. *L.* *petauri* sp. nov. 159469^T^ (NCBI accession number KY548925) is presented in bold type.

The genome of the *L. petauri* sp. nov. 159469^T^ was sequenced on an Illumina MiSeq platform with 2×250 bp paired-end reads, which were assessed for quality with FastQC version 0.11.2 and assembled *de novo* with SPAdes version 3.6.2 [14]. Samtools version 1.3.1 [15] and quast version 3.2 [16] were used to confirm a high quality of the draft genome (e.g. 319× average coverage, 33 contigs >1 Kb, 2.4 Mb total length, N50 of 352908). The assembled genome of *L. petauri* sp. nov. 159469^T^ and genomes of seven type strains of species and subspecies of the genus *Lactococcus* extracted from NCBI were analysed using kSNP version 2 with kmer size 19 [17] to identify core genome single-nucleotide polymorphisms (SNPs). Core genome SNPs (*N*=109) were used to reconstruct a maximum-likelihood phylogeny with 1000 bootstrap repetitions in RaxML (Fig. 2; [11]). Based on the core genome SNPs, *L. petauri* sp. nov. 159469^T^ clustered close to *L. garvieae* JCM 10343^T^; the divergence between these two strains was robust, as demonstrated by a high bootstrap value of 81.

**Fig. 2.**
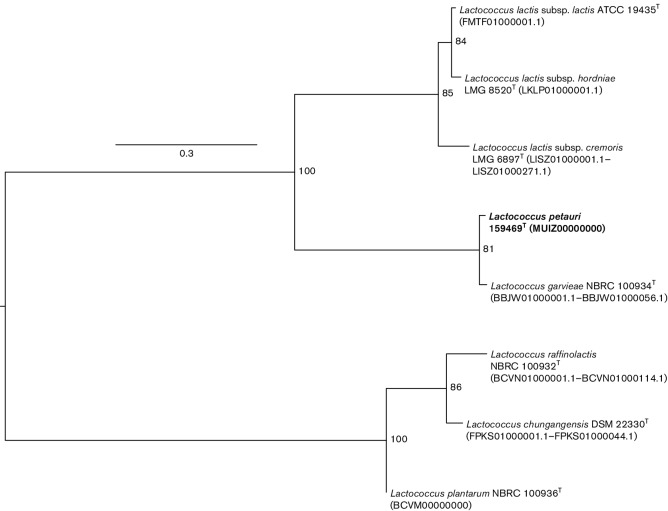
Core genome single-nucleotide polymorphism (SNP) based maximum-likelihood tree including *Lactococcus petauri* sp. nov. 159469^T^ and seven type strains of species and subspecies of the genus *Lactococcus*. The tree was reconstructed with the general time-reversible substitution model and 1000 bootstrap repetitions in RAxML using core genome SNPs identified by kSNP. The tree was rooted by midpoint. Numbers at nodes indicate percentage bootstrap support. Bar, 0.3 substitutions per site. *L. petauri* sp. nov. 159469^T^ (NCBI accession numbers SRR5220185 and MUIZ00000000) is presented in bold type.

The same eight genomes were used to compute pairwise average nucleotide identity blast (ANIb) (https://github.com/widdowquinn/scripts/blob/master/bioinformatics/calculate_ani.py). An ANIb pairwise similarity matrix was used to plot the dendrogram in R 3.3.2 [18] using the ‘hclust’ method (Fig. 3). *L. garvieae* JCM 10343^T^ was shown to have the most similar genome (93.54 %) to *L. petauri* sp. nov. 159469^T^ as suggested by ANIb. The pairwise ANIb values <95 % compared with representatives of other species in the genus *Lactococcus* confirmed strain 159469^T^ as a representative of a novel species [19]. Interestingly, the pairwise ANIb value between *L. petauri* sp. nov. 159469^T^ and another strain, currently classified as a representative of *L. garvieae* in the NCBI database (strain PAQ102015-99, BioSample accession number SAMN04958039) was 98.51 %. This indicates the existence of another strain of the novel species described here as *L. petauri* sp. nov. Strain PAQ102015-99 was described as a putative pathogen of salmonid fish used for vaccine development.

**Fig. 3.**
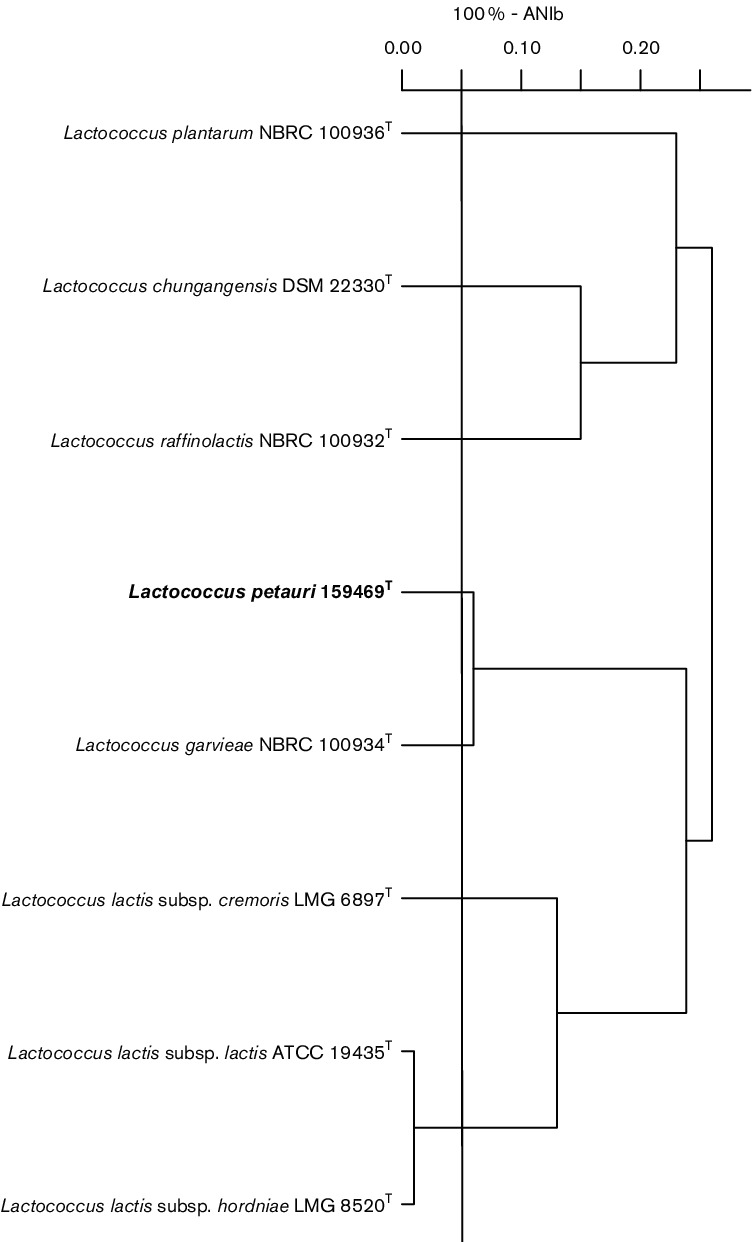
Pairwise average nucleotide identity blast (ANIb) values for *Lactococcus petauri* sp. nov. 159469^T^, and seven type strain of species and subspecies of the genus *Lactococcus*. ANIb values are presented as 100 % – ANIb. The horizontal line indicates the 95 % ANIb species cut-off [19].

*In silico* DNA–DNA hybridization (DDH) analysis was performed on the aforementioned eight genomes using GGDC 2.1 method 2, which is recommended for draft genomes (http://ggdc.dsmz.de/distcalc2.php). The highest DDH value (DDH=50.7 %) was obtained for isolates *L. petauri* sp. nov. 159469^T^ and *L. garvieae* JCM 10343^T^. Considering DDH of 70 % as a species threshold, *in silico* DDH further confirmed isolate 159469^T^ as a representative of a novel species (Table 1; [20, 21]).

**Table 1.** *In silico* computed DNA–DNA hybridization values for *L. petauri* sp. nov. 159469^T^ and seven species and subspecies of the genus *Lactococcus*

Query genome	Reference genome*	DDH†	Model CI (%)‡	Bootstrap CI (%)‡	Distance	Probabaility DDH ≥70 %	DNA G+C content difference (mol%)
Isolate 159469^T^	*L. garvieae* NBRC 100934^T^	50.7	(48–53.3)	50.7–50.7	0.0701	20.98	0.83
Isolate 159469^T^	*L. lactis* subsp. *cremoris* LMG 6897^T^	22.8	(20.6–25.3)	22.8–22.9	0.1918	0	2.18
Isolate 159469^T^	*L. chungangensis* DSM 22330^T^	22.3	(20–24.7)	22.2–22.3	0.1968	0	0.96
Isolate 159469^T^	*L. lactis* subsp. *lactis* ATCC 19435^T^	22.2	(20–24.7)	22.2–22.2	0.1973	0	2.47
Isolate 159469^T^	*L. lactis*subsp.*hordniae* LMG 8520^T^	22	(19.8–24.5)	22–22	0.1991	0	2.88
Isolate 159469^T^	*L. raffinolactis* NBRC 100932^T^	21.4	(19.2–23.9)	21.4–21.5	0.2048	0	2.06
Isolate 159469^T^	*L. plantarum* NBRC 100936^T^	20.6	(18.4–23)	20.6–20.7	0.213	0	0.97

*Lactococcus petauri sp. nov. 159469^T^, MUIZ00000000; *L. garvieae* NBRC 100934^T^, BBJW01000001.1; *L. lactis* subsp. *cremoris* LMG 6897^T^, LISZ01000001.1; *L. chungangensis* DSM 22330^T^, FPKS01000001.1; *L. lactis* subsp. *lactis* ATCC 19435^T^, FMTF01000001.1; *L. lactis*subsp.*hordniae* LMG 8520^T^, LKLP01000001.1; *L. raffinolactis* NBRC 100932^T^, BCVN01000001.1; *L. plantarum* NBRC 100936^T^, BCVM01000001.1.

†Value computed using GGDC 2.1, method 2.

‡CI, credible interval.

To confirm phylogenetic distinctiveness of *L. petauri* sp. nov. 159469^T^, the *rpoB* sequence was extracted from the whole-genome sequence. It was then aligned with *rpoB* sequences of 14 type strains of species and subspecies of the genus *Lactococcus* for which *rpoB* sequences of sufficient length were available in the NCBI database. This alignment was used to reconstruct a neighbour-joining tree with the Tamura 3-parameter substitution model and 1000 bootstrap repetitions in mega 6.0 (Fig. 4; [12]). Clustering of *L. petauri* sp. nov. 159469^T^ based on *rpoB* phylogeny was consistent with that based on 16S rRNA gene phylogeny.

**Fig. 4.**
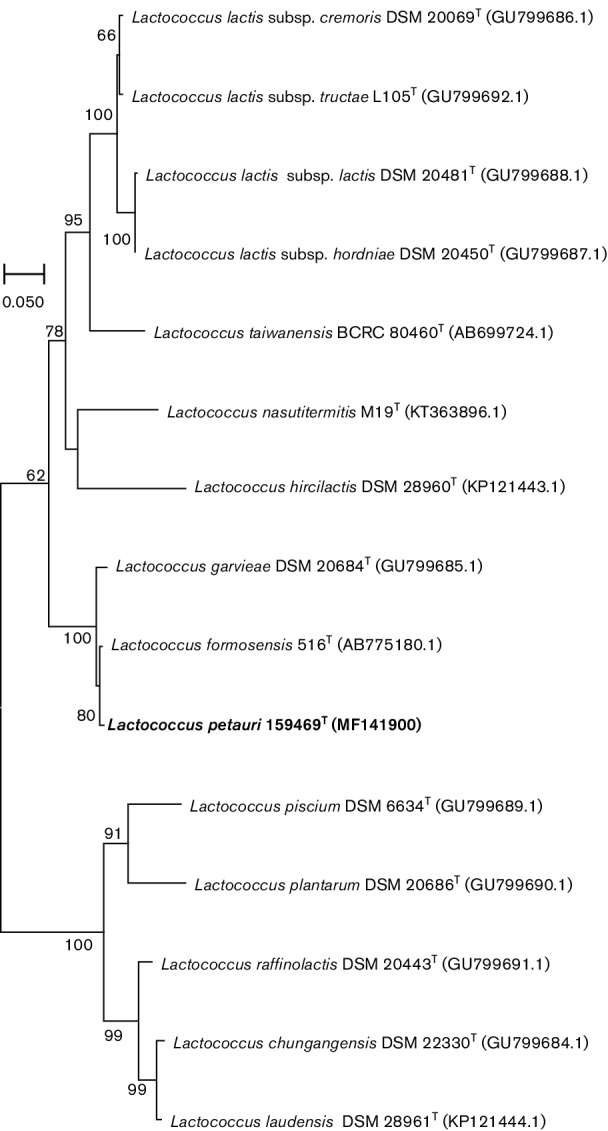
Rooted *rpoB* neighbour-joining tree of *Lactococcus petauri* sp. nov. 159469^T^, 15 type strains of species and subspecies of the genus *Lactococcus*. The tree was reconstructed in mega version 6.0, using the Tamura 3-parameter model and 1000 bootstrap repetitions. Bar, 0.05 substitutions per site. *Lactococcus petauri* sp. nov. 159469^T^ (NCBI accession number MF141900) is presented in bold type.

Strain 159469^T^ was isolated from a lesion on the chin of a 2-year-old female sugar glider at the North Dakota State University Veterinary Diagnostic Laboratory in 2016. The lesion was described as an abscess that was not responding to enrofloxacin. A beta-haemolytic, mucoid and pigmented member of the genus *Lactococcus* was isolated at 4+, which was interpreted as ‘heavy growth’. Alpha-haemolytic streptococci and non-haemolytic staphylococci were also present in moderate numbers. A Gram stain was performed on the abscess swab, and low numbers of Gram-stain-negative rods and Gram-stain-positive cocci were present. This mixed growth was typical from an abscess, but the representative of the genus *Lactococcus* was the predominant colony type. Gram staining from those colonies revealed Gram-stain-positive cocci that were catalase-negative. No identification could be obtained using the biochemical profile on the Sensititre platform (Thermo Scientific). The Biolog and matrix-assisted laser desorption/ionization time-of-flight (MALDI-TOF) platforms both ranked *L. garvieae* as the closest match with scores of 0.734 and 2.1. The strain was propagated on trypticase soy agar (TSA) with 5 % Sheep Blood (BD) at 33–37 °C and demonstrated growth typical of a facultative anaerobe.

Further phenotypic characterization was performed for *L. petauri* sp. nov. 159469^T^ using *L. garvieae* ATCC 43921^T^ as a control. M17 medium (BD Difco) was used for all tests except where indicated differently. Strains were incubated at 30 °C except where indicated differently. Colonies grown on M17 agar were small, round and cream-coloured when incubated aerobically for 24 h at 30 °C. However, colonies appeared larger and orange-coloured when incubated anaerobically for 24 h at 30 °C on M17 agar. *L. petauri* sp. nov. 159469^T^ was oxidase-negative (Hardy Diagnostics) and produced acid and no gas from glucose (BD BBL).

Growth at various temperatures was determined by plating overnight cultures on M17 agar plates and incubating at 4, 6, 10 and 14 °C for 21 days, 20 and 25 °C for 14 days, 30, 35, 37 and 40 °C for 7 days, and 45 and 55 °C for 3 days. *L. petauri* sp. nov. 159469^T^ was able to grow at temperatures of between 6 and 40 °C, but not at 45 °C. Growth at various pH levels was assessed by inoculating pH-adjusted M17 broth and incubating at 30 °C for 14 days. The novel species was able to grow from pH 4.0 to 10.0. Tolerance to various sodium concentrations was determined by inoculating M17 broth containing 3, 4, 5, 6, 7 and 8 % (w/v) NaCl and incubating at 30 °C for 14 days. The novel species was able to grow with 3 to 7 % (w/v) NaCl and no growth was seen at 8 % (w/v) NaCl. Results of the temperature, pH and NaCl tests can be seen in Table 2 compared with those of other type strains of the genus *Lactococcus*.

**Table 2.** Phenotypic properties of *L. petauri* sp. nov. 159469^T^ and type strains of species and subspecies of the genus *Lactococcus* with validly published names Strains: 1, *L. petauri* sp. nov. 159469^T^; 2, *L. garvieae* ATCC 43921^T^; 3, *L. formosensis* 516^T^; 4, *L. fujiensis* NJ 317^T^; 5, *L. chungangensis* CAU 28^T^; 6, *L. piscium* DSM 6634^T^; 7, *L. plantarum* DSM 20686^T^; 8, *L. raffinolactis* DSM 20443^T^; 9, *L. lactis* subsp. *cremoris* KCCM 40699^T^; 10, *L. lactis*subsp.*hordniae* KCTC 3768^T^; 11, *L. lactis* subsp. *lactis* KCTC 3769^T^. Data from strains 1 and 2 are from this study. Data from strains 3–11 are as indicated. +, Positive activity; −, no activity; w, weakly positive activity; nd, no data.

Characteristic	1	2	3^a^*	4*^b^*	5*^c^*	6*^c^*	7*^c^*	8*^c^*	9*^c^*	10^*c*^	11*^c^*
Growth at:											
4 °C	−	−	nd	nd	+	+	−	−	−	−	−
10 °C	+	+	−	+	+	−*^b^*	−*^b^*	−*^b^*	−*^b^*	−*^b^*	−*^b^*
40 °C	+	+	nd	−	−	−	−	−	−	−	+
pH 5.0	+	+	−	+	−*^b^*	−*^b^*	−*^b^*	−*^b^*	+*^b^*	w*^b^*	−*^b^*
pH 10.0	+	−	nd	nd	nd	nd	nd	nd	nd	nd	nd
Growth with 4 % NaCl	+	+	+	nd	−	−	+	−	−	−	+
Growth with 6 % NaCl	+	+	+	−	−*^b^*	−*^b^*	−*^b^*	−*^b^*	−*^b^*	−*^b^*	−*^b^*
Acid from:											
d-Ribose	+	+	+	+	−*^b^*	−*^b^*	−*^b^*	−*^b^*	−*^b^*	−*^b^*	−*^b^*
d-Xylose	−	−	−	−	−	+	−	+	+	−	+
d-Galactose	+	+	+	nd	−	+	+	w	+	−	+
d-Mannitol	w	+	+	+	+*^b^*	−*^b^*	+*^b^*	−*^b^*	−*^b^*	−*^b^*	−*^b^*
Methyl α-d-mannopyranoside	−	−	−	+	−	+	−	−	−	−	−
Methyl α-d-glucopyranoside	−	−	−	nd	−	+	+	−	−	−	w
Amygdalin	+	+	+	−	+	+	+	−	−	−	+
Maltose	+	+	+	nd	+	+	+	+	+	−	+
Lactose	−	−	−	nd	−	+	−	−	+	−	+
Melibiose	−	−	−	nd	−	+	−	+	+	−	−
Sucrose	+	−	−	nd	+	+	+	+	+	+	−
Trehalose	+	+	+	nd	w	+	+	+	+	+	+
Melezitose	−	−	−	nd	−	+	+	−	−	−	−
Raffinose	−	−	−	nd	−	+	−	+	+	−	−
Starch	−	−	−	+	w*^b^*	−*^b^*	−*^b^*	w*^b^*	−*^b^*	−*^b^*	w*^b^*
Gentiobiose	+	+	+	+	w*^b^*	w*^b^*	+*^b^*	w*^b^*	−*^b^*	−*^b^*	w*^b^*
Turanose	−	−	−	nd	w	+	+	−	−	−	−
d-Tagatose	+	−	−	+	nd	nd	nd	nd	nd	nd	nd.
Enzyme activity											
Leucine arylamidase	+	+	+	−	+	+*^b^*	−*^b^*	+^b^	−*^b^*	+*^b^*	w^b^
Acid phosphatase	+	+	+	+	+*^b^*	+*^b^*	+*^b^*	+*^b^*	−*^b^*	+	+*^b^*
β-Glucuronidase	−	−	−	+	−	w*^b^*	w*^b^*	+^b^	−*^b^*	−*^b^*	+^b^

*Data from: *a,* Chen *et al.* [22]; *b,* Cai *et al.* [23]; *c,* Cho *et al.* [24]).

Production of acid from carbohydrates was determined by using API 50 CH kits (bioMérieux). The kits were used according to the manufacturer’s instructions and incubated for 48 h at 30 °C. Enzymic activity was assessed using the API ZYM kit (bioMérieux). The kit was used according to the manufacturer’s instructions. Strains were initially grown aerobically at 30 °C for 24 h on M17 agar. Once the strip was inoculated, incubation occurred for 4 to 4.5 h at 37 °C. The results of the API tests can be seen in Tables 2, S1 and S2.

Analysis of fatty acid methyl esters was performed by Microbial ID according to the instructions of the Microbial Identification System. The organism was cultured on TSA for 24 h at 28 °C before harvesting. The major fatty acids of *L. petauri* sp. nov. 159469^T^ were C_16 : 0_ (40.93 %) and C_14 : 0_ (14.43 %). The complete fatty acid profile of the novel species is shown in Table 3. Comparisons with other species of the genus *Lactococcus* cannot be made due to the use of different incubation conditions by other authors for fatty acid analysis. Fatty acid profiles of other species in the genus can also be seen in Table 3 with the incubation conditions indicated.

**Table 3.** Cellular fatty acid composition of *L. petauri* sp. nov. 159469^T^ and type strains of species and subspecies of the genus *Lactococcus* with validly published names Strains: 1, *L. petauri* sp. nov. 159469^T^; 2, *L. garvieae* KCTC 3772^T^; 3, *L. formosensis* 516^T^; 4, *L. fujiensis* NJ 317^T^; 5, *L. chungangensis* CAU 28^T^; 6, *L. piscium* DSM 6634^T^; 7, *L. plantarum* DSM 20686^T^; 8, *L. raffinolactis* DSM 20443^T^; 9, *L. lactis* subsp. *cremoris* KCCM 40699^T^; 10, *L. lactis* subsp. *hordniae* KCTC 3768^T^; 11, *L. lactis* subsp. *lactis* KCTC 3769^T^. Values represent the percentage of the total fatty acids as determined by the Microbial Identification System software. Data for strain 1 are from this study. Data for the rest of the strains are as indicated. Growth conditions for fatty acid analysis are as follows: this study, TSA agar, 24 h, 28 °C; Chen *et al.* [22], MRS agar, 72 h, 37 °C; Cai *et al.* [23], MRS agar, 48 h, undefined temperature; Cho *et al.* [24], TSA agar, 72 h, 30 °C (except for *L. piscium* – anaerobic *Acetomicrobium faecalis* medium, 72 h, 37 °C). nd, None detected, na, data not available, tr, trace amounts detected.

Fatty acid	1	2^a^*	3*^b^*	4*^c^*	5*^a^*	6*^a^*	7*^a^*	8*^a^*	9*^a^*	10*^a^*	11*^a^*
C_12 : 0_	0.4	4	nd	nd	1.6	nd	1.8	nd	nd	nd	nd
C_14 : 0_	14.43	19.4	5.28	6.1	17.3	nd	10.8	8.2	10	3.4	8.9
C_15 : 0_	nd	0.8	0.42	na	nd	nd	nd.	0.4	0.5	0.3	nd
C_16 : 0_	40.93	34.6	22.73	16.6	37.6	nd	51.1	26.7	40.3	32.6	45.7
C_17 : 0_ cyclo	0.4	nd	0.15	na	nd	nd	nd	0.5	0.7	nd	nd
C_17 : 0_	0.69	na	na	na	na	na	na	na	na	na	na
C_18 : 0_	1.7	0.9	2.95	1	1.2	nd	2.1	0.7	0.7	1.3	2.7
11-Methyl C_18 : 1_ω7*c*	0.57	nd	0.65	na	nd	nd	nd	1.9	1.9	nd	0.5
C_19 : 0_ cyclo ω8*c*	9.41	nd	17.95	na	nd	nd	nd	43.8	31.5	nd	12.5
C_20 : 2_ω6,9*c*	0.45	nd	na	na	nd	nd	nd	1.3	1.5	tr	nd
Summed feature 3†	10.01	na	na	na	na	na	na	na	na	na	na
Summed feature 8‡	21.01	na	na	na	na	na	na	na	na	na	na

*Data from :*a*, Cho *et al.* [24]; *b,* Chen *et al.* [22]; *c*, Cai *et al.* [23].

†Summed feature 3 indicates percentage for C_16 : 1_ ω6c and C_16 : 1_ ω7c.

‡Summed feature 8 indicates percentage for C_18 : 1_ ω6c and C_18 : 1_ ω7c.

## Description of *Lactococcus petauri* sp. nov.

*Lactococcus petauri* (pe.tau′ri. N.L. gen. n. *petauri* of *Petaurus* pertaining to the sugar glider *Petaurus breviceps*).

Cells are Gram-stain-positive cocci, catalase-negative, oxidase-negative, beta-haemolytic, mucoid and facultatively anaerobic. The type strain is orange-pigmented when grown aerobically on TSA with 5 % sheep blood or when grown anaerobically on M17 agar; cream coloured when grown aerobically on M17 agar. It grows at 6–40 °C, but not at 4 or 45 °C; it grows with 7 % (w/v) NaCl and at pH 4.0–10.0, but not at pH 3.0. The organism grows optimally at 20–40 °C, between pH 6.0 and 7.0, and at NaCl concentrations of 3 % NaCl or lower. Produces acid from d-ribose, d-galactose, d-glucose, d-fructose, d-mannose, *N*-acetylglucosamine, amygdalin, arbutin, aesculin, salicin, cellobiose, maltose, sucrose, trehalose, gentobiose and d-tagatose; to some degree also from d-mannitol and potassium gluconate. Possesses active esterase, esterase lipase, leucine arylamidase, α-chymotrypsin, acid phosphatase, α-glucosidase and β-glucosidase, and weakly active valine arylamidase and naphthol-AS-BI-phosphohydrolase. The major fatty acid is C_16 : 0_.

The type strain, isolated from a sugar glider in the USA, is 159469^T^ (=LMG 30040^T^=DSM 104842^T^). The genomic DNA G+C content of the type strain, determined on the basis of the whole-genome sequence, is 37.7 mol%.

## Supplementary Data

322 Supplementary File 1
