# Supplementary material for: Lactococcus petauri sp. nov., isolated from an abscess of a sugar glider
Source: Int J Syst Evol Microbiol. 2017 Sep 25;67(11):4397–404. doi: 10.1099/ijsem.0.002303 (PMC5845659; doi:10.1099/ijsem.0.002303)

Table S1: Carbohydrate fermentation profiles of *L. petauri* 159469$^T$ and *L. garvieae* ATCC 43921$^T$

| | *L. petauri* 159469$^{T}$ [a] | *L. garvieae* ATCC 43921$^{T}$ [a] |
|---|---|---|
| Control | - | - |
| Glycerol | - | - |
| Erythritol | - | - |
| D-Arabinose | - | - |
| L-Arabinose | - | - |
| D-Ribose | + | + |
| D-Xylose | - | - |
| L-Xylose | - | - |
| D-Adonitol | - | - |
| Methyl-BD-Xylopyranoside | - | - |
| D-Galactose | + | + |
| D-Glucose | + | + |
| D-Fructose | + | + |
| D-Mannose | + | + |
| L-Sorbose | - | - |
| L-Rhamnose | - | - |
| Dulcitol | - | - |
| Inositol | - | - |
| D-Mannitol | w | + |
| D-Sorbitol | - | - |
| Methyl-AD- | - | - |
| Methyl-AD-Glucopyranoside | - | - |
| N-AcetylGlucosamine | + | + |
| Amygdalin | + | + |
| Arbutin | + | + |
| Esculin | + | + |
| Salicin | + | + |
| D-Cellobiose | + | + |
| D-Maltose | + | + |
| D-Lactose | - | - |
| D-Melibiose | - | - |
| D-Saccharose | + | - |
| D-Trehalose | + | + |
| Inulin | - | - |
| D-Melezitose | - | - |
| D-Raffinose | - | - |
| Amidon (starch) | - | - |
| Glycogen | - | - |
| Xylitol | - | - |
| Gentiobiose | + | + |
| D-Turanose | - | - |
| D-Lyxose | - | - |
| D-Tagatose | + | - |
| D-Fucose | - | - |
| L-Fucose | - | - |
| D-Arabitol | - | - |
| L-Arabitol | - | - |
| Potassium Gluconate | w | w |
| Potassium 2-KetoGluconate | - | - |
| Potassium 5-KetoGluconate | - | - |

Data obtained from API 50 CH test kit.

[a] +, positive; –, negative; w, weakly positive.

Table S2: Enzyme profiles of *L. petauri* $159469^T$ and *L. garvieae* ATCC $43921^T$

| | *L. petauri* $159469^{T\ c}$ | *L. garvieae* ATCC $43921^{T\ c}$ |
|---|---|---|
| Control | 0 | 0 |
| Alkaline phosphatase | 0 | 0 |
| Esterase (C4)[a] | 3 | 3 |
| Esterase lipase (C8) | 3 | 3 |
| Lipase (C14) | 0 | 0 |
| Leucine arylamidase | 3 | 4 |
| Valine arylamidase | 0 | 1 |
| Cystine arylamidase | 0 | 0 |
| Trypsin | 0 | 0 |
| α-chymostrypsin | 1 | 1 |
| Acid phosphatase | 2 | 2 |
| Napthol-AS-BI-phosphohydrolase[b] | 1 | 1 |
| α-galactosidase | 0 | 0 |
| β-galactosidase | 0 | 0 |
| β-glucuronidase | 0 | 0 |
| α-glucosidase | 2 | 2 |
| β-glucosidase | 1 | 0 |
| N-acetyl-β-glucosaminidase | 0 | 0 |
| α-mannosidase | 0 | 0 |
| α-fucosidase | 0 | 0 |

Data obtained via API ZYM kit (BioMérieux).
[a]Esterase test gave a positive reaction for both organisms but there was a note from the manufacturer that quality control has revealed a lack of sensitivity for this test.
[b]Napthol-AS-BI-phosphohydrolase test gave a slight positive (1) in reagent control (*P. aeruginosa* ATCC 27853™) when it should have been negative.
[c]Color intensity: 0 = no activity, 5 = high activity.

Figure S1: *Lactococcus petauri* 159469$^{T}$ on blood agar after 24 hrs (a) or 48 hrs (b, c, d) of aerobic growth.

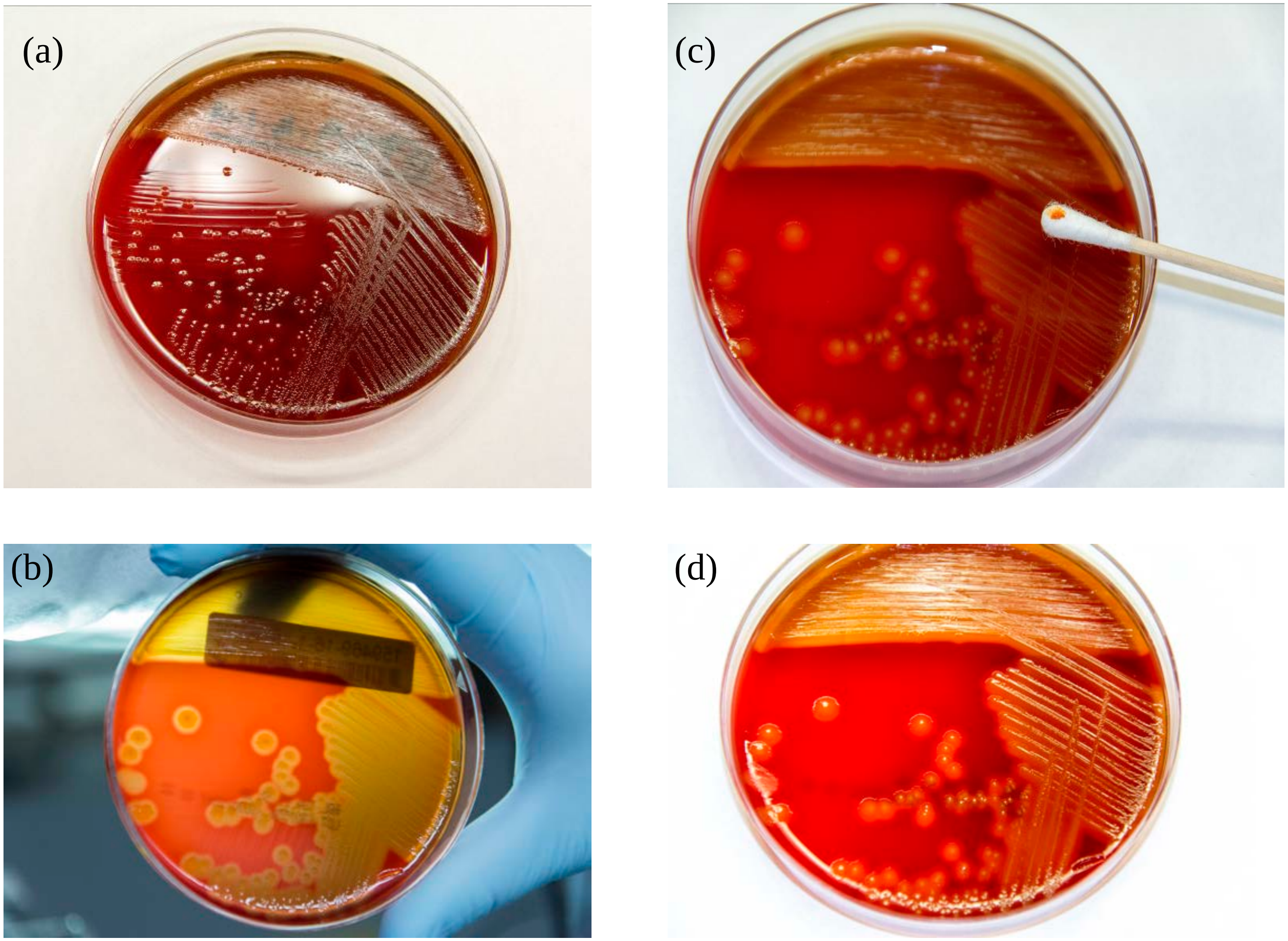

Figure S2: 16S rRNA neighbor-joining tree

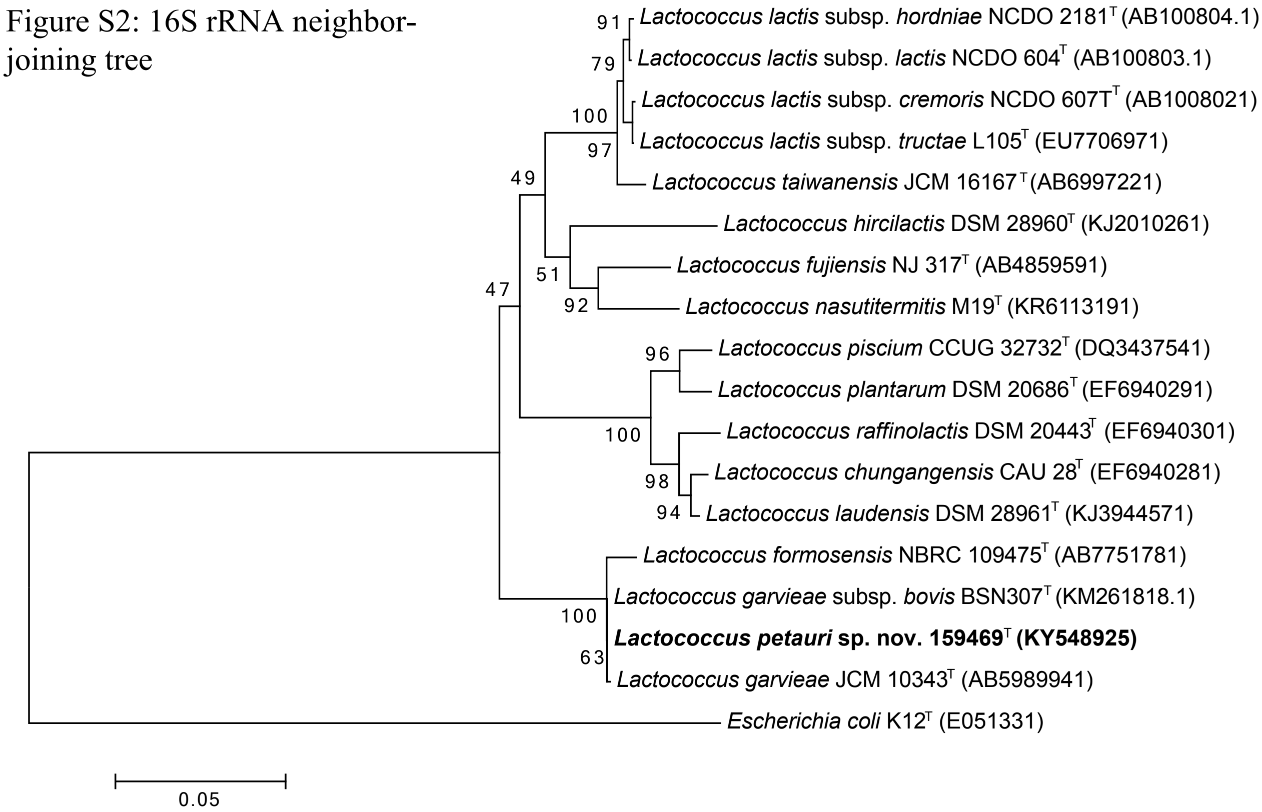

Figure S3: 16S rRNA maximum parsimony tree

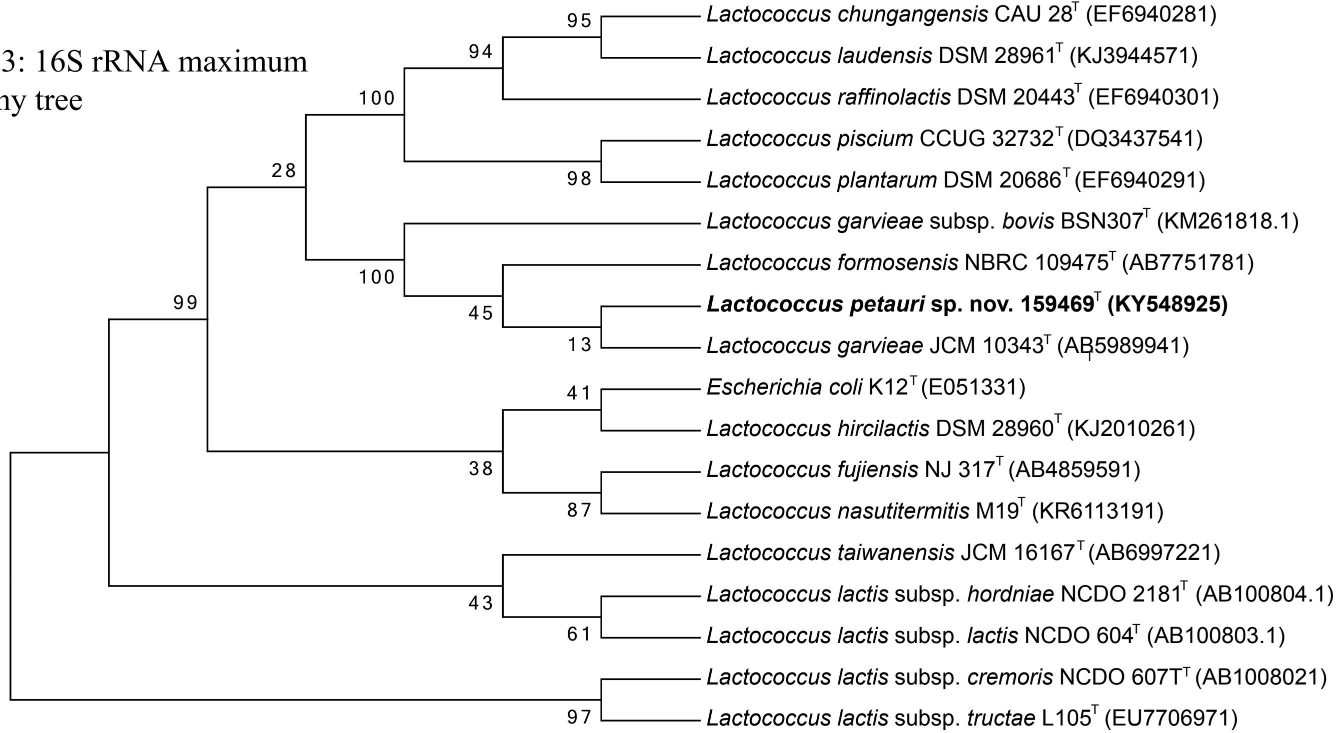

Supplement: Supplementary File 1 [file ijsem-67-4397-s001.pdf]
